# Lethal *Aeromonas veronii* Sepsis in the Course of Medicinal Leech Therapy

**DOI:** 10.3390/antibiotics11091180

**Published:** 2022-08-31

**Authors:** Christoph Sproll, Julian Lommen, Adriana Balasiu, Lara Schorn, Norbert R. Kübler, Birgit Henrich, Rainer Kram, Sabine Petersdorf

**Affiliations:** 1 Department of Oral and Maxillofacial Surgery, Medical Faculty, University Hospital of the Heinrich-Heine-University, 40225 Düsseldorf, Germany; 2 Institute of Medical Microbiology and Hospital Hygiene, Medical Faculty, University Hospital of the Heinrich-Heine-University, 40225 Düsseldorf, Germany; 3 Department of Anesthesiology, University Hospital of the Heinrich-Heine-University, 40225 Düsseldorf, Germany; 4 Institute for Medical Laboratory Diagnostics, Helios University Hospital Wuppertal, 42283 Wuppertal, Germany

**Keywords:** reconstructive surgery, venous flap congestion, leech therapy, *Aeromonas* spp., 16S rDNA sequencing, randomly amplified polymorphic DNA (RAPD)

## Abstract

A patient with oral squamous cell carcinoma (OSCC) underwent complex surgical tumor therapy, including the reconstruction of soft tissues using a radial forearm flap. Due to venous congestion that could only partly be resolved by revision surgery, leech therapy was started on the second postoperative day. The patient developed pneumonia and sepsis and died as a result of septic shock, despite having received targeted broad-spectrum antibiotic therapy since day 5. *Aeromonas* spp. were cultured from both the patient’s specimens and unused leeches. Biochemical identification and matrix-assisted laser desorption ionization time-of-flight mass spectrometry (MALDI-TOF MS) yielded inconsistent identification results. Finally, microbiological identification of *Aeromonas* spp. was performed via 16S rDNA sequencing and use of the basic local alignment search tool (BLAST), and strains from both the patient and the leeches were identified as *Aeromonas veronii*. *Aeromonas* spp. strains derived from the patient and leeches and independent laboratory strains were submitted to randomly amplified polymorphic DNA (RAPD) subtyping. RAPD of *A. veronii* strains from both sources revealed an identical pattern, strongly suggesting the transmission of *A. veronii* from the leeches to the patient. Physicians should be aware of the potential for severe lethal infections as a fatal side-effect of leech therapy in critically ill patients, which should be addressed using antibiotic prophylaxis.

## 1. Introduction

Leeches are one of the oldest medical treatments of mankind. Their ability to suck blood from the patient painlessly yet effectively made their use more popular than mechanical methods of bloodletting, such as the fleam and scarifier, even in the days of Galen’s humoral pathology [1]. Shortly after the discovery of blood circulation [2], many diseases nonetheless continued to be attributed to an excess of blood in the body [1], and the consumption of leeches continuously increased and peaked in the 19th century, with an annual consumption of 100 million [3]. The advancement of scientific medicine, especially its subfields of physiology and pathology, eventually led to the decline of this centuries-old application [4].

Scientific interest in leeches resurged when F. Markwardt isolated hirudin, which has a very strong anticoagulant effect, from leech pharyngeal glands in 1955 [5]. Thus, leeches moved into focus in the context of plastic surgery, where they were used to treat venous congestion of flaps, beginning in 1960 and continuing thereafter [6].

Based on the discovery of hirudin, the mechanism of action and the constituents of leech saliva were subjected to intensive basic research. Although it was previously assumed that the saliva was only used for sucking [1], it has now been possible to identify and characterize a large number of adjuvant ingredients of the saliva, which serve the goal of an undisturbed blood meal over 15–30 min and subsequent painless phlebotomy. These include substances that inhibit coagulation (hirudin, calin, saratin, destabilase, and apyrase), have analgesic and anti-inflammatory effects (eglin C and leech-derived tryptase inhibitor, LDTE), facilitate tissue penetration via degradation of the extracellular matrix (hyaluronidase and collagenase), and have vasodilatory (antistasin) or antimicrobial effects; for reviews, see [7,8]. It has been possible to define useful indications through several well-founded case series and to develop recommendations for use and algorithms for leech application with regard to dosage per area, delivery route, frequency of application, and appropriate antibiotic prophylaxis [9]. For example, leeches are now used to prevent flap necrosis in congested pedicled or free flaps in plastic surgery [8] and in replantation surgery in the replantation of various body parts [7]. Other applications of leeches are in orthopedics, in the treatment of osteoarthritis, in the treatment of chronic regional pain syndromes, and otitis media [10]. The risks of infection, which can hardly be controlled in free-living animals, had already led to the institutionalization and regulation of leech farming at the beginning of the 19th century (with the foundation of Biopharm^®^ in Hendy, South Wales, UK, in 1812), with the aim of making their application as safe as possible. This culminated in their approval as a medical device by the US Food and Drug Administration (FDA) in 2004 [11] and for approval as a ready-to-use drug in Germany by the Federal Institute for Drugs and Medical Devices (BfArM) in 2005 [12]. As with other drugs, there are also risks and side effects associated with the use of leeches, which must be carefully considered before use. These include, first and foremost, severe blood loss, which in extreme cases can lead to the need for a transfusion [13] or even death [14]. Furthermore, other risks include regional lymph node swelling, local itching, allergic reactions to protein components of leech saliva such as local pruritus and anaphylaxis (grade I–IV) [15,16], and the development of B-cell pseudolymphoma [17].

One of the greatest risks, however, is due to the potential for infections, which may present as narrowly localized or systemic inflammation caused by secondary infections of the bite site or primarily through the transmission of the leeches’ intestinal symbionts, *Aeromonas* spp. [18]. Moreover, the occurrence of inflammatory complications significantly lowers the success rate of flap salvage [13,19]. In addition, there is a risk that the inflammation will spread, causing the patient to become critically ill. Thus, there are reports in the literature of septic courses of *Aeromonas* infection [20], but so far, these have invariably had ultimately favorable outcomes. In this respect, we report here on the first fatal course of such a systemic spread of infection.

## 2. Materials and Methods

The diagnostic laboratory of the Institute of Medical Microbiology and Hygiene of the University Hospital Düsseldorf is accredited according to DIN EN ISO 15198:2014. Blood culture diagnostics: blood culture bottles were incubated in an automated system (BacT/Alert^®^, bioMérieux, Nürtingen, Germany) for 7 days. Processing of tracheal secretion samples: samples were homogenized with dithiothreitol (DDT) and streaked on Columbia blood agar plates (bioMérieux, Nürtingen, Germany), MacConkey agar plates (Oxoid, Munich, Germany) and chocolate agar plates (bioMérieux, Nürtingen, Germany) and incubated for 48 h in aerobic conditions for MacConkey plates, with additional 5% CO_2_ pressure at 36 °C for Columbia blood agar plates or for 96 h in anaerobic conditions at 36 °C for chocolate agar plates. Pathogen identification was carried out by means of matrix-assisted laser desorption ionization time-of-flight mass spectrometry (MALDI-TOF MS) technique using the Vitek^®^MS mass spectrometer (bioMérieux, Nürtingen, Germany). Antibiotic resistance testing was performed using the Vitek^®^2-System (bioMérieux, Nürtingen, Germany).

**Figure 1:**
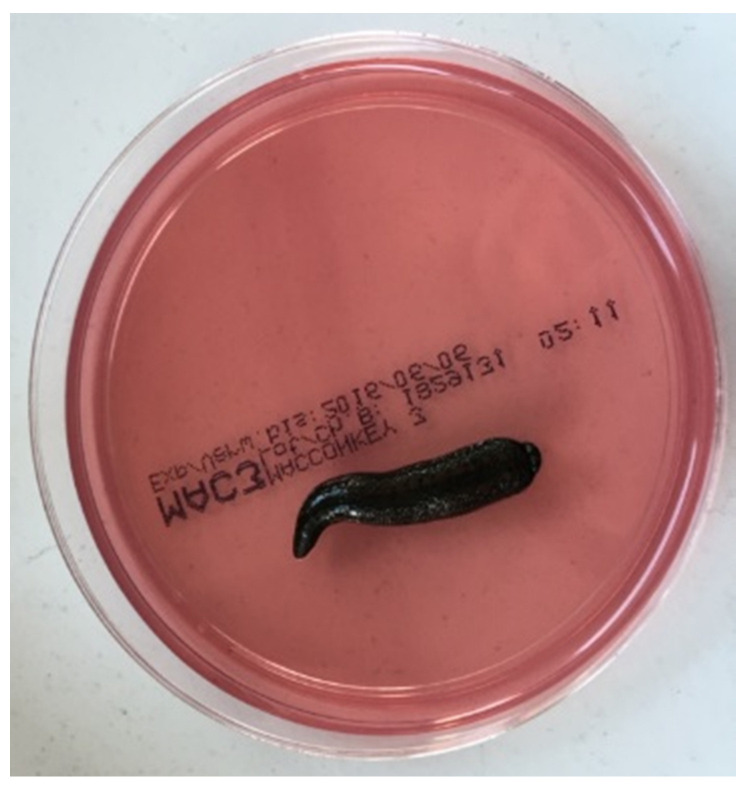
Leech on MacConkey Agar Plate.

Microbiological cultures from leeches (method not accredited): Bacteria were grown from live leeches by placing the leeches directly on Columbia blood agar plates (bioMérieux, Nürtingen, Germany) and MacConkey agar plates (Oxoid, Munich, Germany) for 2 min (Figure 1). Plates were then incubated for 18 h at 36 °C. All the bacterial species growing on the plates were subcultured and submitted to Vitek^®^MS and Vitek^®^2 for identification and antibiotic resistance testing, respectively.

Sequencing of 16S rDNA and comparison to databases: DNA extraction was performed using a BioRobot EZ1^®^ (Qiagen, Hilden, Germany), 16S rDNA real-time PCR, and melt curve analysis, which was carried out on an iCycler^®^ (Bio-Rad, Munich, Germany), and the sequencing of PCR products was performed in the Biomedical Research Facility of the Medical Faculty of the Heinrich-Heine University, Düsseldorf. Sequence comparison was carried out using the basic local alignment search tool (BLAST) (http://blast.ncbi.nlm.nih.gov/Blast.cgi, accessed on 22 November 2016). A characterized bacterial reference strain was simultaneously analyzed with 16S rDNA PCR and melting curve analysis as a positive control.

Randomly amplified polymorphic DNA (RAPD) was performed as described elsewhere [21] using four primers: AP40, ST272, Eric1R, and Eric2 (Eurofins Genomics^®^, Ebersberg, Germany). The following *Aeromonas* spp. strains were subtyped by RAPD:

- *Aeromonas veronii* isolate from blood culture;
- Two *Aeromonas veronii* isolates cultured from leeches from the same container in which the leeches used for the patient were kept;
- Four *Aeromonas hydrophila* isolates from leeches from a different container;
- Two independent *Aeromonas hydrophila* isolates derived from unrelated patients in terms of time and place.

## 3. Case Presentation

**Figure 2:**
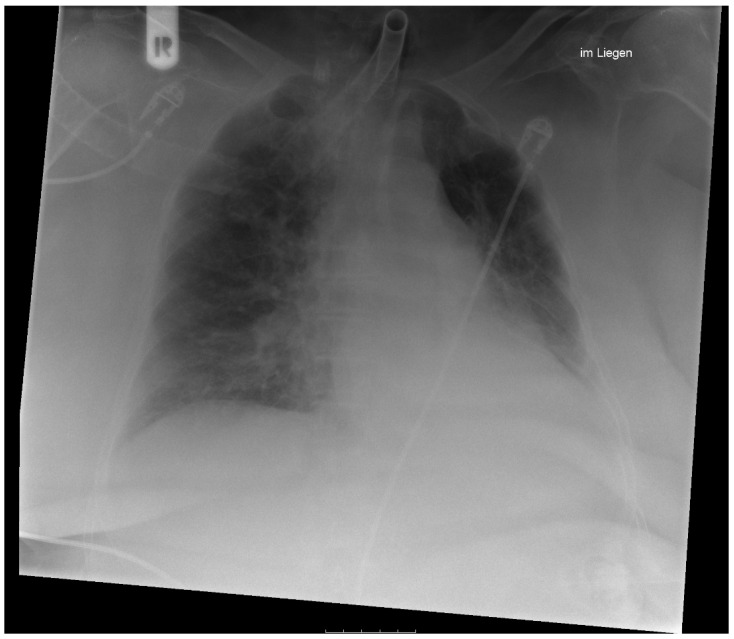
Chest X-ray from the fourth postoperative day: Twisted image with lateralization of the mediastinum to the left. Inserted tracheal cannula, the tip of which projects approximately 6.5 cm cranially to the carina onto the tracheal lightening band. The left dome of the diaphragm is not sharply delineated. Marked areal compression left-retrocardially. Prominent pulmonary hili on both sides as well as somewhat blurred vascular markings. In the supine position, no evidence of a pneumothorax. Assessment: Compaction retrocardially on the left side, consistent with a pneumonic infiltrate. Mild PV congestion.

A 75-year-old Caucasian female patient presented at our clinic with multilocular p16-negative oral squamous cell carcinoma (OSCC). Three independent intraoral manifestations were detectable. The patient reported a lifelong abstinence from alcohol and tobacco. The patient underwent complex tumor surgery according to the decision made by the head and neck tumor board. For pre-existing medical conditions and detailed information on the location and staging of OSCC, see Table 1. Due to venous congestion of the radial forearm flap on the first postoperative day, revision surgery was performed with the removal of a venous thrombus and revision of the venous anastomosis. Due to insufficient flap recovery, two leeches were placed on the flap three times a day until spontaneous detachment. The breeding leeches (*Hirudo verbana*) were obtained from a national certified breeding farm (Medirud^®^, Biebertal, Germany). Antibiotic therapy with 4.5 g piperacillin/tazobactam (three times daily) was administered when the patient developed pneumonia on the fifth postoperative day (Figure 2) and meropenem at 1 g three times daily was included from day 6, when her condition deteriorated (Table 2). The patient died from sepsis with multiorgan failure on day 7. Microbiological examination of tracheal secretion and blood cultures revealed the presence of *A. veronii*.

## 4. Results

### Microbiologic Cultures

Patient: *A. veronii* was grown from the tracheal secretion and blood cultures. The pathogens of both locations showed the following results in antibiotic resistance testing: ampicillin/amoxicillin R ≥ 32, ampicillin + sulbactam R ≥ 32, piperacillin I 16, piperacillin + tazobactam S ≤ 4, cefuroxime S ≤ 1, ceftazidime S ≤ 1, ertapenem S ≤ 0.5, gentamicin S ≤ 1, ciprofloxacin S ≤ 0.25, moxifloxacin S ≤ 0.25, and TMP/SMZ (Co-Trim) S ≤ 20; minimum inhibitory concentration (MIC) determination: meropenem (mg/L) S 0.06, imipenem (mg/L) S 0.5, and cefotaxime (mg/L) S 0.03.

Leeches: As expected, *Aeromonas* spp. was isolated from all the examined leeches. In addition, further gram-negative rods, including *Citrobacter werkmannii*, *Stenotrophomonas maltophilia*, *Comamonas testeroni*, and *Pseudomonas fluorescens*, were isolated from the leeches. Since identification using the Vitek card AST-N223 and MALDI-TOF MS returned discrepant results (*A. sobria* and *A. veronii* resp.), *Aeromonas* strains from blood cultures, and from leeches from the same container as those used on the patient, and from leeches from an independent container, were submitted to 16S rDNA sequencing. The blood culture strain and the strain from the leeches used on the patient were both identified as *Aeromonas veronii*, whereas strains from the leeches taken from the independent container were identified as *Aeromonas hydrophila*.

**Figure 3:**
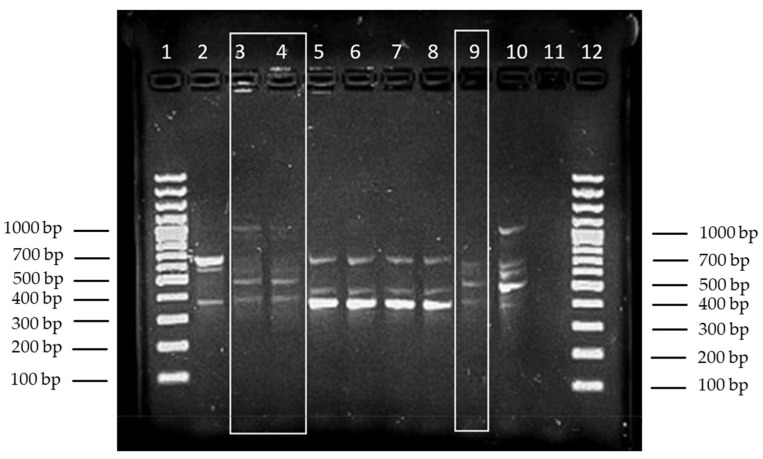
Results for the subtyping of different *Aeromonas* spp. strains using RAPD PCR with Eric2 primers. *Aeromonas veronii* strains from blood culture (column 9) and from leeches of the same container as those used on the patient (columns 3 and 4) showed an identical pattern. *Aeromonas hydrophila* strains from 4 leeches from a different container (columns 5–8) showed a pattern identical to each other but a different to that of the patient strain. *Aeromonas hydrophila* strains from independent patients showed independent patterns (columns 2 and 10). Columns 1 and 12 are lambda ladders (GeneRuler 100 bp DNA ladder, Thermo Fisher Scientific^®^, Waltham, MA, USA) used as size markers.

RAPD typing showed that *Aeromonas* spp. from leeches from the same container had the same RAPD patterns. Strains isolated from blood cultures and those from the unused leeches in the same container showed an identical pattern and are thus genetically related. In contrast, *Aeromonas* strains from a different container, although related to each other, were revealed to be unrelated to those of the patient and those from independent laboratory strains (Figure 3). This strongly suggests that *Aeromonas veronii* were transmitted from the leeches to the patient.

## 5. Discussion

We report here the first case of lethal sepsis caused by *A. veronii* during leech therapy due to venous congestion of a radial forearm free flap. We assume that there is a causal relationship because the patient showed a clear constellation of infection with a massive increase in infection parameters, a classic inflammatory/septic circulatory failure (see Table 1) and we were not able to detect any pathogen other than *A. veronii* in the laboratory samples of sputum and blood. Via RAPD typing, we were able to show that the germs from the leeches had been transferred to the patient. The aeromonads from the leeches from the container were genetically identical to those from the blood culture.

The microbiome of wild *H. verbana* contains a large spectrum of pathogen species of various genera ranging from *Morganella*, *Clostridia*, *Erysipelothrix*, and *Desulfovibrio* to *Fusobacteria* and is dominated by two species, *A. veronii* and *Mucinivorans hirudinis* [10]. *A. veronii* is an obligate intestinal symbiont that is vertically inherited and assists the leech in blood digestion through the ability to β-hemolyze [22]. In the directions for the use of these leeches, tightly localized inflammation, associated sometimes with papular efflorescence at the bite sites and often with pruritus, is listed as a common risk (>1/100), whereas marked local inflammation, e.g., cellulitis, erysipelas, phlegmon, or lymphangitis, is listed as rare (>1/10,000), and systemic infections with sepsis, e.g., via secondary infection of the wound by different pathogens or by primary infection with *A. hydrophila* or *A. veronii* biovar sobria, are listed as very rare (<1/10,000) [12]. Finally, inflammatory complications have led to the standardization and regulation of leech farming and to the approval of leeches in the United States as a medical device in 2004 [23] and in Germany as a ready-to-use drug in 2005.

In this respect, by definition, only leeches in which the feeding blood is free of viral human pathogens and that have completed a minimum 32 week feeding-free quarantine may be shipped in Germany. Microbiological testing prior to shipment must ensure that no human pathogens (e.g., pathogens of the genera *Clostridium*, *Staphylococcus*, *Streptococcus*, *Listeria*, and *Pseudomonas* and species of Enterobacteriaceae such as *E. coli*, or *Klebsiella* spp.) other than *Aeromonas* spp. are detectable on the skin or in the leech internal organs. Most gut symbionts of *H. medicinalis* are not culturable, necessitating the use of DNA-based methods for germ detection and differentiation [23]. Likewise, the transport medium must be free of the abovementioned pathogens [12]. Accordingly, we were only able to detect *A. veronii* in the tracheal secretions and blood culture of our patient. However, in the leeches themselves, we found other gram-negative bacteria such as *Citrobacter werkmannii*, *Stenotrophomonas maltophilia*, *Comamonas testeroni*, and *Pseudomonas fluorescens*.

There are four other case reports of septic complications of leech therapy in the literature to date: in these cases, as in the present case, no antibiotic prophylaxis was received [20] or it was ineffective [24,25]. Evans et al. reported septic complication after infection with *A. hydrophila* and *Enterobacter* following the reestablishment of backflow from the hand after an occupational injury to the right hand. When leeches were applied, the patient received cephradine and metronidazole, which proved ineffective [24]. Levine et al. reported on a patient with venous insufficiency after replantation of the thumb and revascularization of fingers 2–4. General prophylaxis with ampicillin–sulbactam was continued during leech therapy. After the arrival of findings with evidence of ampicillin–sulbactam-resistant *A. veronii* biovar sobria, the antibiotic was changed to ciprofloxacin, and the patient’s condition rapidly improved thereafter [25]. Maetz et al. reported on two patients with delayed breast reconstruction via a pedicled TRAM flap. In the first patient, the flap had to be repositioned because of venous congestion, and the patient received leech therapy for 4 days. She then developed a septic infection due to *A. veronii* and received IV antibiotics with 1.5 g vancomycin/day and 6 g cefotaxime/day for 10 days and 350 mg amikacin/day for 2 days. The fever improved after 4 days of this antibiotic treatment. The other patient was also subjected to two-stage breast reconstruction with a pedicled TRAM flap. Because of venous congestion, she was treated with leeches during postoperative days 2–5. On day 8, necrosectomy had to be performed, and 4 g amoxicillin/clavulanic acid/day and 160 mg gentamicin/day were initially given for 2 days to treat septic infection. After the arrival of the microbiology findings, with evidence of *A. veronii* and the corresponding resistogram, the administered antibiotics were changed to 6 g cefotaxime/day for 10 days, followed by 400 mg ofloxacin p.o./day for 8 days. In this case, the fever again decreased after three days [20]. The flap success rates of leech therapy of 10 case series were documented in a recent meta-analysis by Mousavian et al. The courses of 298 patients could be evaluated, and the success rate for partial or complete salvage was 60.73%; 16.77% of flaps were lost, and the fate of another 22.5% of patients was unknown [8]. Inflammatory complications can develop within the first 24 h (as in our case) or up to a month later [9]. This is thought to be due to secondary spread from necrotic, locally immunocompromised areas [26]. In cases in which inflammatory complications developed, the rate of flap salvage was more than halved across all case series, such as from 88.3% to 37.4% (in this series, 94% *Aeromonas* spp., mostly *A. hydrophila*, were the causative pathogens) [13] or from 83% to 31.8% [19]. In our case, the flap showed signs of recovery at the time of the patient’s death, but it was not yet possible to definitively predict whether it would heal.

There are no guidelines on the use of leeches based on prospective randomized clinical trials (RCTs) nor evolving risk–benefit profiles [27], but there are recommendations for use. For example, in Germany, the Federal Institute for Drugs and Medical Devices (BfArM) advocates that the need for concomitant AB therapy should be decided on an individual basis. Thus, 3rd gen. cephalosporins or gyrase inhibitors should be used [12]. The literature currently consists of reports and case series relating to a few hundred patients and systematic reviews of these cases [13]. The literature reports frequencies of inflammatory complications of 4.7% [3] of cases with AB prophylaxis and 2.4–20% in patients without appropriate antibiotics [28]. In such cases, the causative bacteria are mostly *Aeromonas* spp. (88%) [27]. Accordingly, the majority of SOPs derived from the current literature recommend antibiotic prophylaxis starting at the beginning of leech therapy and continuing for one week thereafter [10] but are still based in part on data obtained prior to the strict regulation of leech manufacturing and marketing in the United States (2004) and Germany (2005).

Surveys of plastic surgeons indicate that most use AB prophylaxis, and many use effective prophylaxis. Whitaker et al. surveyed all 62 plastic surgery clinics in England and Ireland about leech therapy practices and received feedback from 50. Of these, 37 (74%) used antibiotic prophylaxis with augmentin (30%), metronidazole (5%), benzyl penicillin and/or flucloxacillin (14%), ciprofloxacin (22%), and various (24%) [10].

*A. veronii* can carry different types of beta-lactamases and is always resistant to ampicillin. A variable number of strains are susceptible to ampicillin–sulbactam, and resistance to carbapenems and ciprofloxacin is rare but increasing. Thus, resistance has been increasingly reported in the literature since 2000: Marden et al. reported seven cases from 2011 to 2014 involving limb replantation or flap surgery in which ciprofloxacin-resistant *A. hydrophila* strains were cultured [23]. A study by Wilmer et al. examined 21 *Aeromonas* isolates from 26 leech water samples and found that 15 (71.4%) were sensitive to ciprofloxacin, 13 (61.9%) were sensitive to gentamycin, and 21 (100%) were sensitive to sulfamethoxazole–trimethoprim [29]. Whitaker examined three *Aeromonas* isolates and found that all were resistant to amoxicillin, two were resistant to augmentin, and all were sensitive to ciprofloxacin [30].

In our case, the resistogram was as expected from the literature: Pip/Taz was used here when pathogens were detected and supplemented with meropenem the following day, but this was no longer sufficient.

Pre-therapeutic resistance testing of leeches from the same lot used for therapy would help by facilitating the application of a targeted antibiotic and, thus, not encourage the breeding of further resistance [31]. This is not yet routinely performed in Germany but should be introduced, not least because of this report.

To sum up our findings, infections must be considered as a potentially lethal risk in leech therapy. Leeches must be thoroughly rinsed before therapy, and the application site must be disinfected. They must not be manipulated with a sharp instrument, such as forceps. Regurgitation by pulling off or sprinkling with saline solution should be avoided [27]. Postoperative hemorrhage should not be prematurely stopped. AB prophylaxis is recommended, ideally based on the resistogram of *Aeromonas* spp. strains from other leeches of the same lot.

## 6. Conclusions and Limitations

Regarding this case report and data from meta-analyses, a general recommendation of antibiotic prophylaxis with 3rd generation cephalosporins or gyrase inhibitors during leech therapy is advocated.

## Figures and Tables

**Table 1:** Patient and Tumor Characteristics.

**General medical condition**
Pre-existing medical conditions Metabolic syndrome, including obesity with a BMI of 40.8 (87 kg/146 cm), arterial hypertension, and insulin-dependent diabetes mellitus type II; Chronic renal failure (1.3 mg/dL serum creatinine); Subcortical arteriosclerotic encephalopathy; Bilateral gonarthrosis. No allergies.
**Medication**
Aspirin, valproate, ramipril, insulin, and ranitidine.
**Clinical presentation of tumor disease (OSCC)**
Three independent manifestations: (i) Measuring 2 cm × 1.5 cm on the right side of the lower lip mucosa; (ii) Measuring 4 cm × 1.5 cm on the right buccal plane region 045–048; (iii) 2 cm × 1 cm on the alveolar process of the mandible region 037–038.
**Tumor Surgery**
Tracheostomy; partial resection of the mandible, floor of the mouth, tongue, cheek, and lower lip; and bilateral selective neck dissection. Reconstruction of the mandibular bone by means of a mandibular reconstruction plate and of the oral soft tissues using a tongue flap and radial forearm free flap.
**Final TNM-stage of the tumors**
The final TNM-stage was (i) pT2 pN0(0/33) L0 V0 Pn1 G3 R0 for the right lower lip; (ii) pT4a pN0(0/33) L0 V0 Pn0 G2 R0 for the alveolar process of the right mandible; (iii) pT2 pN0(0/33) L0 V0 Pn0 G3 R1 for the left floor of the mouth.

**Table 2:** Clinical Status and medical intervention Protocol; (P = prophylaxis, T = therapy, TID = 3 times daily).

Day	Intervention	Clinical Symptoms	CRP (mg/dL)	Fibrinogen (mg/dL)	Procalcitonin (ng/mL)	WBC (×1000/µL)	SaO_2_ (%)	Body Temperature (°C)	Pathogen Detection	Antibiotic Prophylaxis (P)/Therapy (T)
1	Initial Tumor Surgery		N/A	N/A	N/A	10.2	98.7	35.6	N/A	Ampicillin/Sulbactam i.v. 3 g 2 times intraoperatively (P)
2	Radial Forearm Flap revision surgery	Venous flap congestion	9.5	308	N/A	9.5	97.5	38.6	N/A	Ampicillin/Sulbactam i.v. 3 g once intraoperatively (P)
3–4	Start of medicinal leech therapy	Venous flap congestion improved but still present	13.8/19.5	366/400	N/A	9.8/11.4	95.2	36.1/38.9	N/A	none
5		Pneumonia	32.9	N/A	0.99	17.5	94.5	39.1	*A. veronii* from tracheal exudate	Piperacillin/Tazobactam 4.5 g TID (T)
6		Septic shock	46.0	663	1.00	18.4	96.8	39.3	*A. veronii* from blood culture	Piperacillin/Tazobactam 4.5 g TID (T)
7		Multiorgan failure, death	48.7	817	5.30	17.5	84.9	39.4		Piperacillin/Tazobactam 4.5 g + meropenem 1 g TID (T)
